# Integrative Taxonomy and Life Stage Associations in *Nectopsyche fuscomaculata* Flint, 1983 (Trichoptera: Leptoceridae)

**DOI:** 10.1007/s13744-026-01406-6

**Published:** 2026-05-29

**Authors:** Pedro Bonfá-Neto, Felipe Ribeiro Pereira Sarmento, Rayner Paresqui Constantino, Roberta Paresque, Frederico Falcão Salles

**Affiliations:** 1 Programa de Pós‑Graduação em Entomologia, Departamento de Entomologia, Universidade Federal de Viçosa (UFV), Viçosa, MG Brazil; 2 Programa de Pós-Graduação Em Biodiversidade Tropical, Universidade Federal Do Espírito Santo (UFES/CEUNES), São Mateus, ES Brazil; 3 Departamento de Ciências da Saúde, Centro Universitário Norte Do Espírito Santo (CEUNES), Universidade Federal Do Espírito Santo (UFES), São Mateus, ES Brazil; 4 Museu de Entomologia, Departamento de Entomologia, Universidade Federal de Viçosa (UFV), Viçosa, MG Brazil

**Keywords:** Aquatic insects, Long-horned caddisflies, DNA Barcode, COI, Semaphoronts

## Abstract

Semaphoronts are entities that carry the characteristics of individuals at different periods over time, essential for understanding species in various biological areas. In the long-horned caddisfly genus *Nectopsyche* Müller, 1879 (Trichoptera: Leptoceridae), there is a significant lack of knowledge about semaphoronts, especially in South America. Historically, descriptions have focused mainly on adult males, with descriptions of larvae, pupae, and adult females occurring in isolated studies. This study addresses the Haeckelian shortfall by associating adults and larvae of the genus *Nectopsyche* using mitochondrial COI gene sequences. The study was conducted in the Rio Doce hydrographic basin, southeastern Brazil, where specimens were collected and photographed. The resulting DNA sequences were compared with data available and analyzed using Neighbor-Joining methods. Our findings show that *N. fuscomaculata* and its congeners were recovered with high support values, presenting a barcode gap of 7% among them. As a result, the association between the adult female and larval semaphoronts with the adult male of *N. fuscomaculata* was established. The previously unknown semaphoronts of this species, namely the female and the larva, are described and illustrated. This study expands current knowledge of the genus’ biological diversity and highlights the importance of integrative taxonomic approaches.

**Supplementary Information:**

The online version contains supplementary material available at 10.1007/s13744-026-01406-6.

## Introduction

Understanding the life stages of species is crucial in several areas of biology, such as phylogenetic reconstructions, studies of morphological structures and their functions, and ecological interactions with the environment and other organisms (Faria et al. 2020). The lack of information of semaphoronts results in a knowledge gap known as the Haeckelian deficit (Faria et al. 2020). An example of the importance of understanding semaphoronts can be found in Trichoptera. This order stands out for the varying levels of sensitivity or tolerance to environmental stress among species, making its members valuable bioindicators for monitoring freshwater ecosystems (Lenat 1993). However, although the larval stage is widely used in biomonitoring, it remains unknown for the majority of species within the order (Pes et al. 2018).

One of the genera within Trichoptera that exhibits a significant Haeckelian deficit is *Nectopsyche* Müller, 1879. This genus is restricted to the Americas, being the most diverse and often the most abundant within the family Leptoceridae in the Neotropical region (Almeida and Marinoni 2000). Studies describing the life stages of *Nectopsyche* focus mainly on species from the Nearctic region (Elkins 1936; Betten and Mosely 1940; Ross 1944; Haddock 1977; Daigle and Haddock 1981; Harris 1986; Wiggins 1996; Schmid 1998; Glover and Floyd 2004), in contrast, only a small fraction of Neotropical species have had their immature stages or females formally described (Flint 1968; Botosaneanu and Flint 1982; Botosaneanu 1994; Holzenthal 1995; Botosaneanu and Hyslop 1998; Holzenthal and Ríos-Touma 2018; Bonfá-Neto and Salles 2023).

Currently, 105 species are recognized in the genus (Assunção and Quinteiro 2023), 90 of which occur in the Neotropical region, 10 in the Nearctic region, and 5 have a Panamerican distribution (Morse 2026). Of these, only two species have all their semaphoronts known (including the pupa), and 13 other species have been described as larvae, females, and males (Table 1). Most of these species occur in North and Central America, with only four distributed in South America (Holzenthal 1995; Haddock 1977; Holzenthal and Ríos-Touma 2018). Regarding species with only two known semaphoronts, seven have associated males and females, four have males and larvae, and one is known only from the male and pupa. In total, based on current knowledge of the life stages of *Nectopsyche* semaphoronts, 27 species have at least two stages described (Table 1).

**Table 1:** Summary of *Nectopsyche* species with associated semaphoronts and their geographic distribution

Species	Biogeographical region	American distribution	Known semaphoronts
*N. albida* (Walker, 1852)	NA	North	M, F, L
*N. aymore* Bonfá-Neto & Salles, 2023	NT	South	M, F
*N. brethesi* (Navas, 1920)	NT	South	M, F
*N. candida* (Hagen, 1861)	NA	North	M, F, L
*N. cubana* (Banks, 1938)	NT	Central	M, F, L
*N. diarina* (Ross, 1944)	NA	North	M, F, L
*N. dorsalis* (Banks, 1901)	NA and NT	Panamerican	M, F, L
*N. exquisita* (Walker, 1852)	NA	North	M, F, L
*N. gemma* (Müller, 1880)	NT	Central and South	M, P
*N. gemmoides* Flint, 1981	NT	Panamerican	M, F, L, P
*N. globigona* Botosaneanu, 1998	NT	Central	M, F, L
*N. gracilis* (Banks, 1901)	NA and NT	North and Central	M, F, L
*N. jenseni* (Ulmer, 1905)	NT	South	M, F
*N. lahontanensis* Haddock, 1977	NA and NT	North	M, L
*N. lewisi* (Flint, 1968)	NT	Central	M, F, L, P
*N. minuta* (Banks, 1900)	NA	North	M, L
*N. muhni* (Navas, 1916)	NT	South	M, F
*N. padrenavasi* Holzenthal, 2000	NT	South	M, F
*N. paludicola* Harris, 1986	NA	North	M, F, L
*N. paramo* Holzenthal & Ríos-Touma, 2018	NT	South	M, F, L
*N. pavida* (Hagen, 1861)	NA and NT	North and Central	M, F, L
*N. quatuorguttata* (Navas, 1922)	NT	South	M, F
*N. spiloma* (Ross, 1944)	NA and NT	Panamerican	M, F, L
*N. splendida* (Navas, 1917)	NT	South	M, F
*N. stigmatica* (Banks, 1914)	NA and NT	North	M, F, L
*N. tavara* (Ross, 1944)	NA	North	M, L
*N. waccamawensis* Glover & Floyd, 2004	NA	North	M, L

Abbreviations: *NA* Nearctic region, *NT* Neotropical region, *M* Male, *F* Female, *L* Larva, *P* Pupa. References: Holzenthal and Calor (2017) and others cited above

Associations between immature stages and adult females of *Nectopsyche* have traditionally relied on methods such as rearing (Wiggins 1996), indirect association (Flint 1974), metamorphotype (Milne 1938), or copulation-based association for females (Queiroz et al. 2021). In recent decades, mitochondrial DNA sequences, especially a fragment of the gene known as cytochrome oxidase I (COI), have been used in taxonomic approaches (Hebert et al. 2003). Since then, DNA barcoding has facilitated the association of life stages in caddisflies, as well as species delimitation and the detection of cryptic species (e.g., Pauls et al. 2010; Ruiter et al. 2013; Santos et al 2016). Nevertheless, this methodology has not yet been applied to the association and delimitation of *Nectopsyche* life stages and species.

In summary, knowledge of *Nectopsyche* species and their life stages is extremely necessary, not only for taxonomic deepening of the group, but also for their use in ecological studies, biomonitoring, and environmental impact assessments (Morse et al. 2019). Therefore, this study aims to expand knowledge of the semaphoronts of the genus *Nectopsyche*, associating adult females and larvae with adult males through COI sequences from sampled specimens. Additionally, it explores species boundaries using genetic distance-based methods.

## Material and Methods

### Sample Collection and Curation

**Fig. 1:**
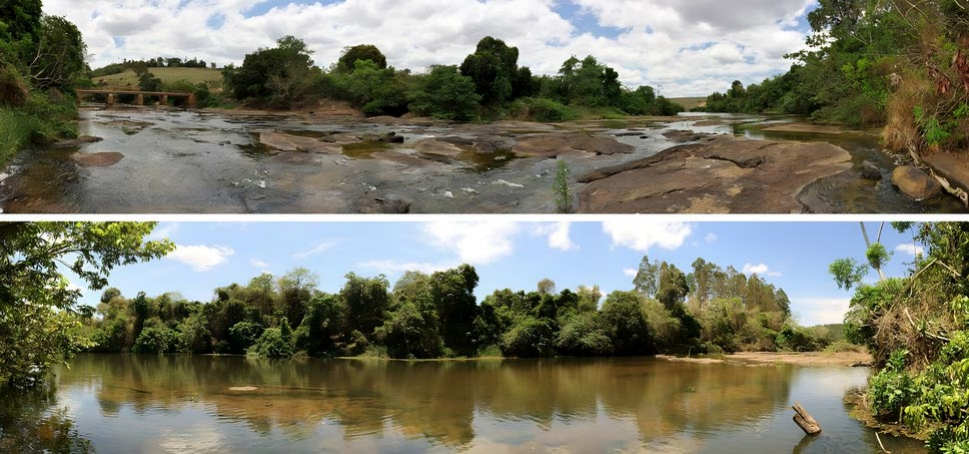
São José River, Sooretama, Espírito Santo state (Brazil). Collection sites for *Nectopsyche* specimens. Panoramic photographs taken by Rayner P. Constantino in 2016

Individuals were collected and analyzed from the São José River (river with significant lentic areas), in the municipality of Sooretama, Espírito Santo state (Fig. 1). Immatures were collected using a D-net or by active manual searching of the substrate (Pes et al. 2019). The *Nectopsyche* larvae were found predominantly along one of the riverbanks, characterized by a high density of marginal grasses partially submerged in the water. This area was shallow with very slow water velocity, characterized by the accumulation of organic matter on the substrate. Adults were collected using Pennsylvania light traps equipped with collecting containers containing ethanol (Frost 1957; Nessimian et al. 2024) which were turned on at dusk and switched off at dawn. The collected material was preserved in absolute ethanol to ensure DNA integrity and deposited in the Museum of Entomology of the Federal University of Viçosa (UFVB).

### Identification and Species Concept

Species identification was based primarily on the male genitalia of males preserved in alcohol, as the color pattern of the body and wing setae were absent. In some specimens, the abdomens were detached and cleared (diaphanized) using 10% KOH (for males and females) and/or 85% lactic acid (males only) (Blahnik and Holzenthal 2004; Blahnik et al. 2007). Subsequently, the genitalia were temporarily transferred to a depression slide containing glycerin gel (approximately 1:1 mixture of RMC Glycerall RF® and PA or bidistilled glycerin) and examined under an optical microscope.

Contemporary species concepts share a common foundation discussed by de Queiroz (2007), who proposed a unified species concept recognizing species as independently evolving metapopulation lineages. Under this unified concept, the properties of distinct species concepts are treated as operational criteria for assessing lineage separation, and multiple independent criteria may be employed (de Queiroz 2007). In the present study, two operational criteria were used to delimit species: (1) diagnosable morphological characters; and (2) genetic distances and base-pair substitution rates of the COI fragment.

### Molecular Data Acquisition

DNA was extracted using one to three legs from each specimen. Samples were subjected to the DNA extraction protocol according to the manufacturer’s instructions using the Promega Wizard® SV Genomic DNA Purification System kit, and were diluted to 100 µL. Subsequently, the DNA products obtained were stored and preserved in freezers at −20 °C. The extracted DNA was amplified by PCR (Polymerase Chain Reaction) targeting the mitochondrial cytochrome oxidase subunit I (COI) region using primers LCO-1490 (5′-GGT CAA CAA ATC ATA AAG ATA TTG G-3′) (Forward) combined with HCO-2198 (5′-TAA ACT TCA GGG TGA CCA AAA AAT CA-3′) (Reverse) (Folmer et al. 1994).

Each PCR reaction consisted of 1 µL of genomic DNA added to 24 µL of a solution containing 2.5 µL of Invitrogen® Buffer MgCl2 10x; 2.5 µL of Invitrogen® MgCl2 50 mM; 0.3 µL of each primer (5 mM); 1 µL of Promega® dNTPs (100 mM); 17.3 µL of ultrapure water (ddH₂O); and 0.10 µL of Invitrogen® Platinum Taq DNA Polymerase. The final volume of 25 µL was subjected to the following thermal cycling conditions: initial denaturation at 94 °C for 5 min, 40 cycles at 94 °C for 45 s, 47 °C for 45 s, 72 °C for 45 s, and a final extension at 72 °C for 5 min. In addition to the desired samples, positive and negative controls were used in all PCRs to check the quality of the added products and possible contamination.

The PCR products underwent electrophoresis in 1% agarose gel to verify amplification success and product quality. PCR products were purified using ExoSap-IT® for PCR Product Cleanup (Invitrogen) at a 1:4 ratio, in which 10 µL of PCR product was added to 1 µL of the diluted ExoSap solution (at a concentration of 1:4), incubated for 30 min at 37 °C followed by 15 min at 80 °C in a thermocycler. Purified samples were sent to Macrogen Inc. (Seoul, South Korea) for sequencing using the LCO-1490 (forward) primer.

### DNA Sequence Analyses

The obtained sequences were subjected to similarity searches using the BLAST tool (Basic Local Alignment Search Tool; http://blast.ncbi.nlm.nih.gov/Blast.cgi). After confirming the fragment identity and taxon, the sequences were analyzed using Geneious v7.1.3 software (Kearse et al. 2012). Sequences were checked for stop codons and double peaks, and inconsistent leading and trailing regions were trimmed. Sequences were subsequently exported in FASTA format. Additionally, to improve resolution, 19 COI sequences from 9 species available in GenBank (Zhou et al. 2016; Hebert et al. 2016) were analyzed together with the sequences generated in this study (Table S1 in Supplementary Material).

In total, 24 specimens (22 adults and two larvae) were processed for DNA extraction and sequencing. From these, 11 high-quality COI sequences were obtained, representing two species of *Nectopsyche* and one of *Oecetis* McLachlan, 1877, all approximately 580 bp in length (Table 2; Table S3 in Supplementary Material). These sequences included seven males, three females, and one larva. Combined with the 19 sequences retrieved from GenBank, a total of 30 COI sequences representing 10 species were analyzed, including one outgroup species.

**Table 2:** Species with COI fragments sequenced in this study, with respective information on registration number, location, life stage, and sex

Species	Voucher	Genbank access code	Locality	Stage	Sex
*Nectopsyche fuscomaculata* Flint, 1983	UFVB TR01082	PX935517	Sooretama, ES, Brazil	Adult	Male
*Nectopsyche fuscomaculata* Flint, 1983	UFVB TR01083	PX935518	Sooretama, ES, Brazil	Adult	Male
*Nectopsyche fuscomaculata* Flint, 1983	UFVB TR01084	PX935519	Sooretama, ES, Brazil	Adult	Female
*Nectopsyche fuscomaculata* Flint, 1983	UFVB TR01085	PX935520	Sooretama, ES, Brazil	Adult	Female
*Nectopsyche fuscomaculata* Flint, 1983	UFVB TR01086	PX935521	Sooretama, ES, Brazil	Adult	Male
*Nectopsyche fuscomaculata* Flint, 1983	UFVB TR01087	PX935522	Sooretama, ES, Brazil	Adult	Male
*Nectopsyche fuscomaculata* Flint, 1983	UFVB TR01088	PX935523	Sooretama, ES, Brazil	Larva	
*Nectopsyche* sp.	UFVB TR01089	PX935524	Sooretama, ES, Brazil	Adult	Male
*Nectopsyche* sp.	UFVB TR01090	PX935525	Sooretama, ES, Brazil	Adult	Male
*Nectopsyche* sp.	UFVB TR01091	PX935526	Sooretama, ES, Brazil	Adult	Male
*Oecetis iguazu* Flint, 1983	UFVB TR01092	PX935527	Sooretama, ES, Brazil	Adult	Female

Sequences were aligned using Clustal W (Thompson et al. 1994), and pairwise genetic distance matrices were calculated in MEGA v11 (Tamura et al. 2021) under the Kimura 2-parameter (K2P) model (Kimura 1980) with pairwise deletion of missing data. Furthermore, a Neighbor-Joining (NJ) tree was constructed to visualize group relationships using K2P distances (Saitou and Nei 1987). Branch support was estimated with 1,000 nonparametric bootstrap replicates (Felsenstein 1985).

### Photographs, Illustrations and Descriptions

The body parts and wings were photographed using a Leica MC170 HD camera attached to a Leica M205 A stereomicroscope, with subsequent editing in Adobe Photoshop CC®. Genitalia were photographed with a Moticam A5 camera (Motic) coupled to an Olympus CX31 microscope; focus stacking was performed using Helicon Focus®. These photographs served as templates for illustrations, which were produced by digitally tracing the structures in Adobe Illustrator CC®. Species descriptions followed the standards established in previous comprehensive descriptions and the revisionary work for the genus (Holzenthal 1995; Holzenthal and Ríos-Touma 2018). The morphological terminology follows Holzenthal (1995) and applies to both adult and immature stages.

### Geographic Distribution Map

The distribution map was created using QGIS version 3.28 'Firenze', employing vector layers in shapefile format from the Brazilian Institute of Geography and Statistics (IBGE) and raster data from Natural Earth. The World Terrestrial Ecosystems layers were obtained from the World Wildlife Fund (WWF) (Olson et al. 2001). Distribution records were compiled from the material examined in this study, data retrieved from the Barcode of Life Data System (BOLD Systems), and records from published literature (Flint 1983; Almeida and Marinoni 2000; Blahnik et al. 2004; Dumas et al. 2009; Dumas and Nessimian 2012; Souza et al. 2013; Dias et al. 2015; Henriques-Oliveira et al. 2019, 2020; Santos et al. 2022; Bonfá-Neto et al. 2023; Sganga et al. 2025; Alvim et al. 2025). Additional information regarding distribution records is provided in the Supplementary Material (Table S2).

## Results

### Genetic Distances and Thresholds

**Fig. 2:**
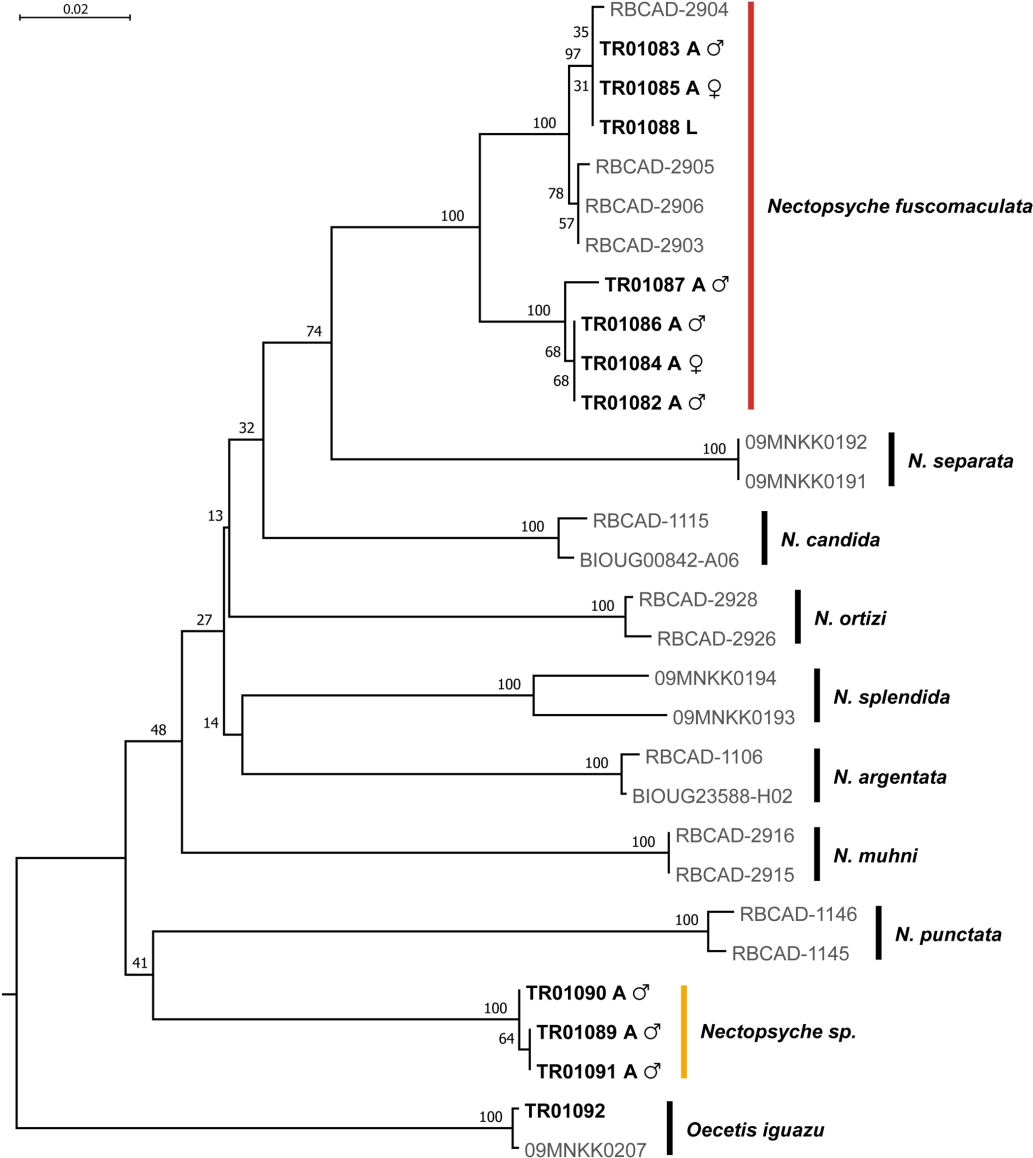
Neighbor-joining tree of COI sequences from *Nectopsyche* species and outgroup based on K2P distances. Voucher in bold = specimens sequenced in this study. Bootstrap support values above the branches

The resulting Neighbor-Joining (NJ) tree showed strong bootstrap support (100%) for all species (Fig. 2). For the outgroup sequences, K2P genetic distances ranged from 19.0% to 21.7% for *Oecetis iguazu*. Within the genus *Nectopsyche*, the maximum intraspecific distance was 4.3%, whereas the minimum interspecific distance was 11.3% (Table 3).

**Table 3:** Intraspecific and interspecific K2P distances of COI sequences for the analyzed species of *Nectopsyche*

Species	N	Max. intra	Min. intra	Max. inter	Min. inter
*Nectopsyche* sp.	3	0.002	0.000	0.204	0.150
*Nectopsyche argentata* Flint, 1991	2	0.003	0.003	0.214	0.118
*Nectopsyche candida* (Hagen, 1861)	2	0.008	0.008	0.223	0.113
*Nectopsyche fuscomaculata* Flint, 1983	11	0.043	0.000	0.212	0.113
*Nectopsyche muhni* (Navás, 1916)	2	0.000	0.000	0.215	0.164
*Nectopsyche ortizi* Holzenthal, 1995	2	0.005	0.005	0.202	0.129
*Nectopsyche punctata* (Ulmer, 1905)	2	0.006	0.006	0.223	0.187
*Nectopsyche separata* (Banks, 1920)	2	0.000	0.000	0.209	0.117
*Nectopsyche splendida* (Navás, 1917)	2	0.043	0.043	0.202	0.128

Abbreviations: *N* Number of sequences analyzed, *max.* Maximum, *min.* Minimum, *intra.* Intraspecific, *inter.* Interspecific

The highest intraspecific distance was recorded for *Nectopsyche fuscomaculata* (K2P = 4.3%). Specimens TR01083, TR01085, and TR01088 exhibited higher similarity to sequences retrieved from GenBank (K2P = 0.0–0.8%) than to specimens TR01082, TR01084, TR01086, and TR01087 from this study (K2P = 3.7–4.3%) (Fig. 2). The *Nectopsyche* sp. specimens yielded Blast Percent Identity values below 90%, suggesting they represent a lineage currently unrepresented in GenBank or potentially a new species. The maximum intraspecific K2P distance was 0.2%, while the minimum interspecific distance was 15% (Table 3).

An unequivocal association was established among all life stages and specimens of *Nectopsyche fuscomaculata* examined in this study, including the larva (TR01088), females (TR01084 and TR01085), and males (TR01082, TR01083, TR01086, and TR01087). Despite the intraspecific genetic variation observed, all individuals clustered together with *N. fuscomaculata* sequences retrieved from GenBank (Fig. 2). Support values for this species-level clade were remarkably high, reaching 100% bootstrap support in the Neighbor-Joining (NJ) tree.

### Taxonomy

Order Trichoptera Kirby, 1813

Family Leptoceridae Leach, 1815

Genus *Nectopsyche* Müller, 1879

Type species: *Setodes gemma* Müller, 1880, by subsequent monotypy by Ulmer (1951).

**Fig. 3:**
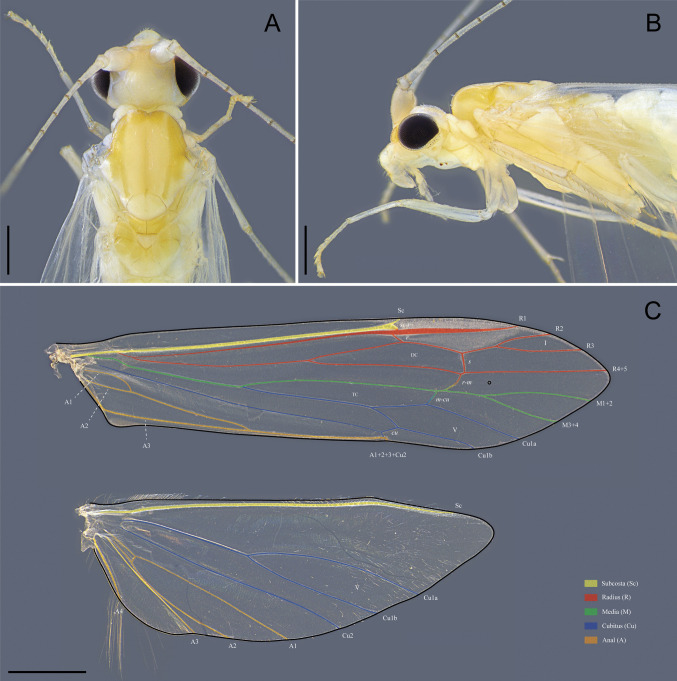
*Nectopsyche fuscomaculata*, female (UFVB TR01085). **A**, head and thorax, dorsal; **B**, head and thorax, lateral; **C**, forewing and hindwing, dorsal. Scale bar: A and B = 0.5 mm; C = 1.0 mm

**Fig. 4:**
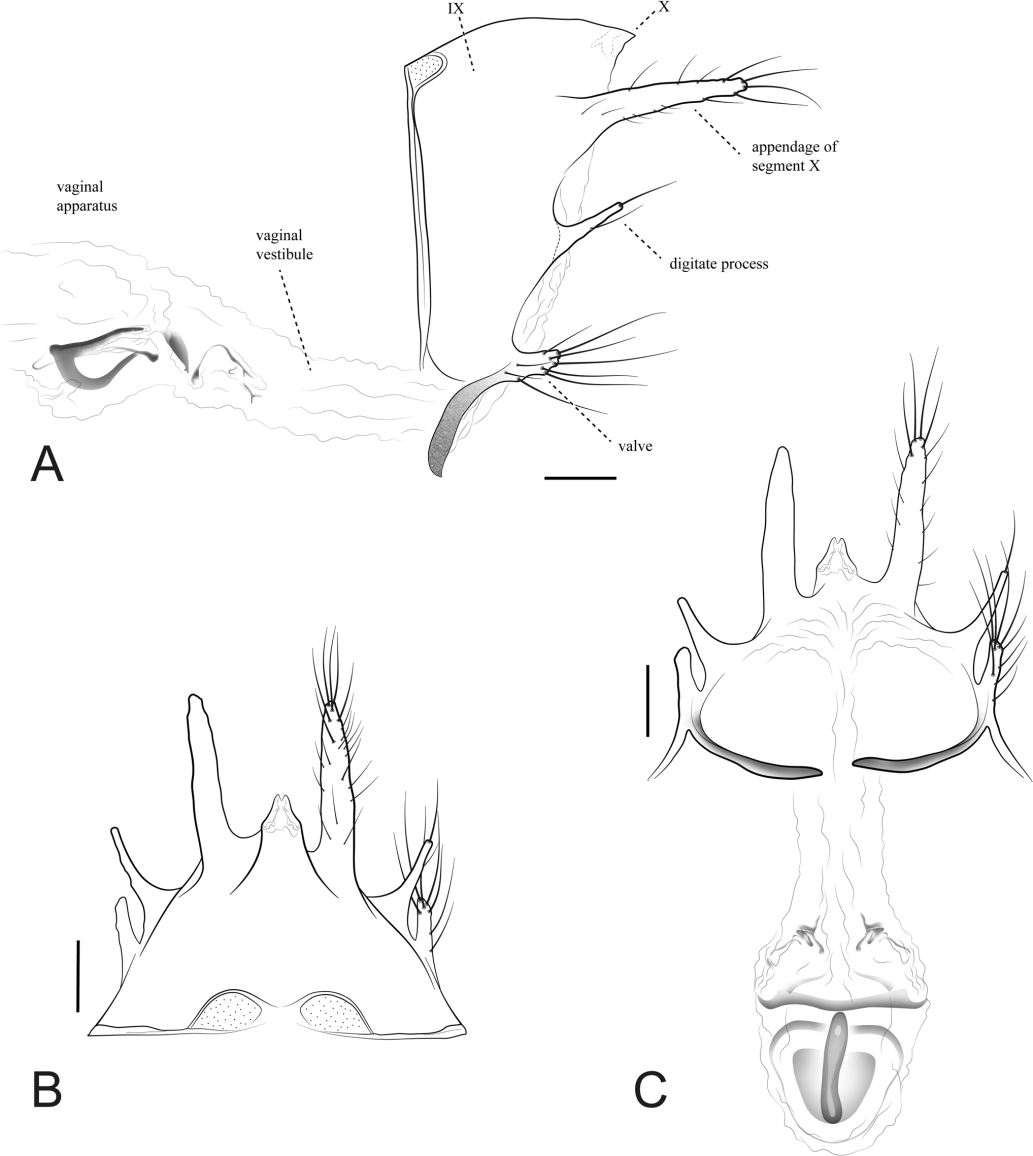
*Nectopsyche fuscomaculata*, female genitalia (UFVB TR01085). **A**, segments IX, X, and vaginal apparatus, ventral; **B**, segments IX and X, dorsal; **C**, segments IX, X, and vaginal apparatus, lateral. Scale bar = 0.1 mm

**Fig. 5:**
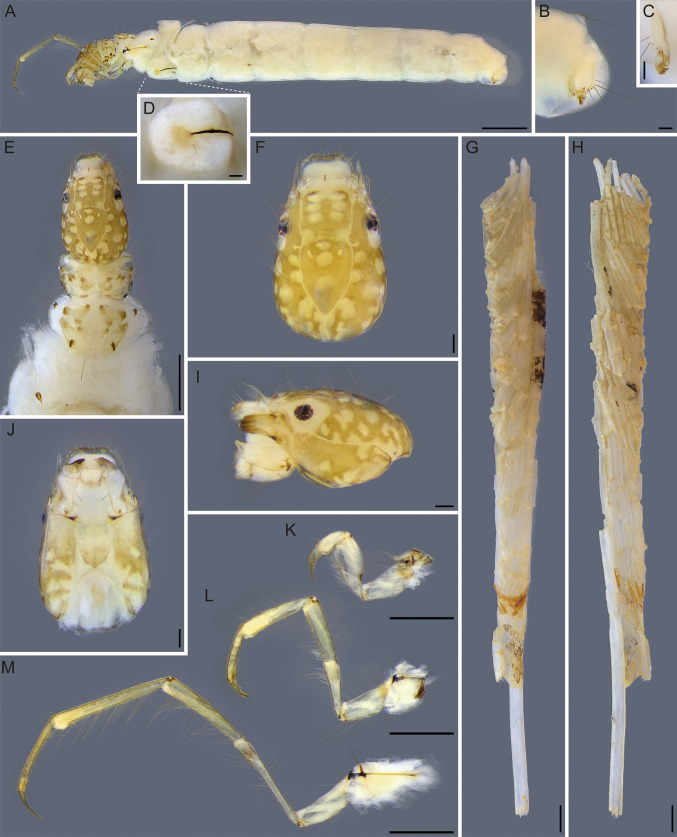
*Nectopsyche fuscomaculata*, larva in alcohol (UFVB TR01088). **A**, habitus, lateral; **B**, abdominal segments IX and X, lateral; **C**, anal proleg and anal claw, posterior view; **D**, lateral hump; **E**, head and thorax, dorsal; **F**, head, lateral; **G**, case, left lateral; **H**, case, right lateral; **I**, head, lateral; **J**, head, ventral; **K**, left foreleg, lateral; **L**, left midleg, lateral; **M**, left hindleg, lateral. Scale bar: A, G, H = 1.0 mm; E, K, L, M = 0.5 mm; B, C, D, F, I, J = 0.1 mm

**Fig. 6:**
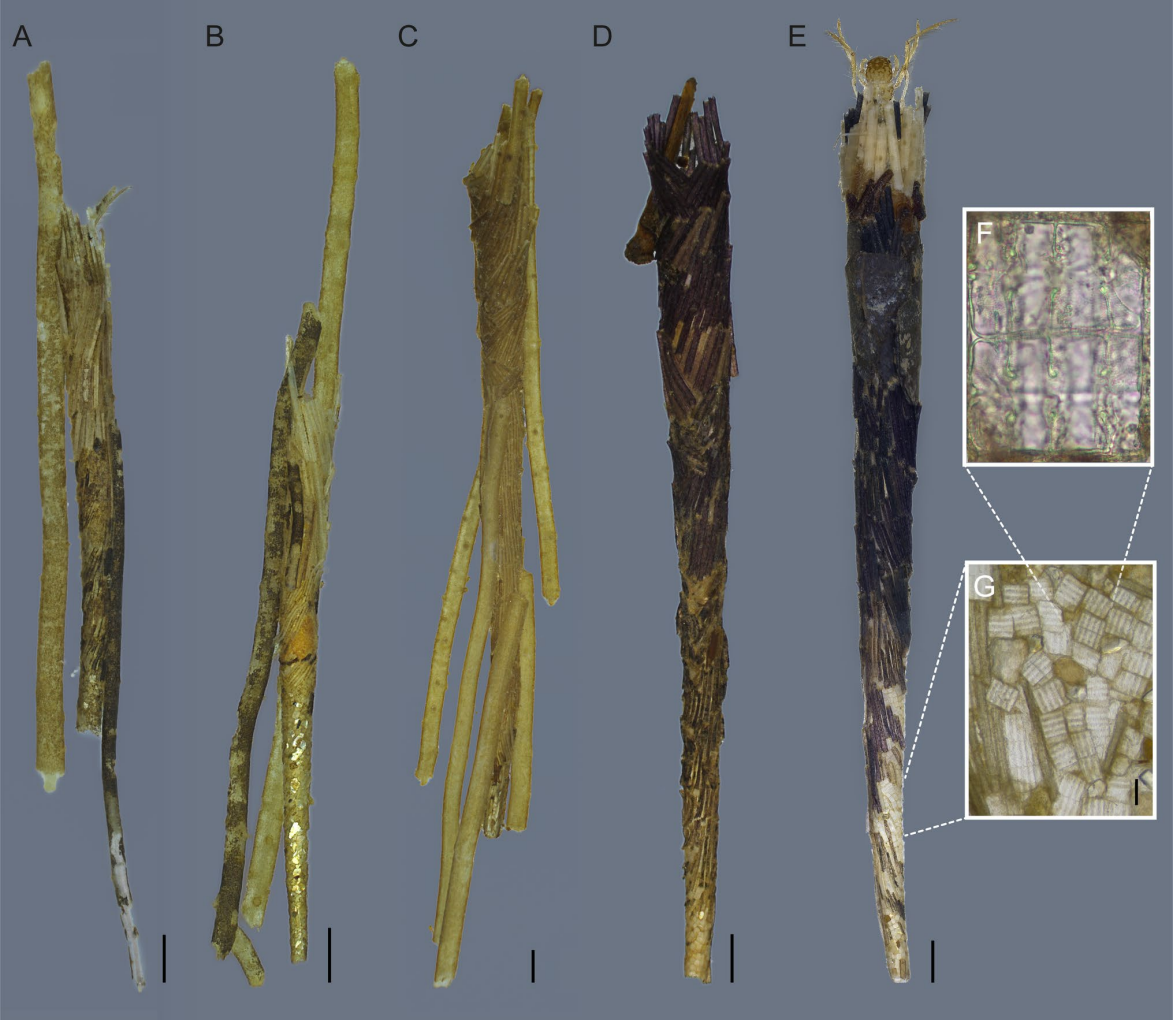
Case of *Nectopsyche fuscomaculata*, in alcohol. **A–E**, case; **F** and **G**, *Terpsinoë* diatoms, in girdle view. Scale bar: A–E = 1.0 mm; F = 0.1 mm

**Fig. 7:**
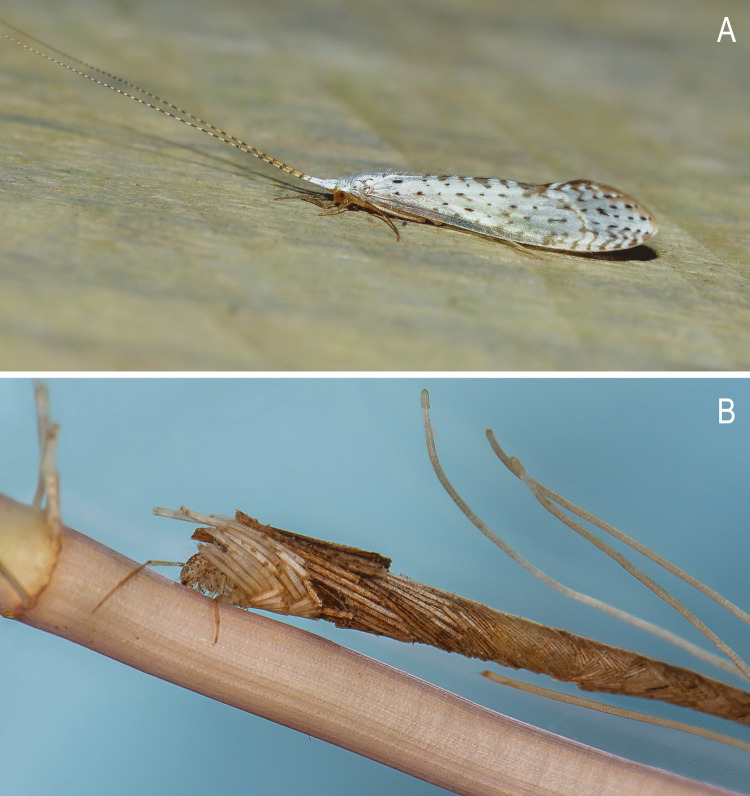
*Nectopsyche fuscomaculata*, live specimens photographed. **A**, adult male, photographed by F.F. Salles, 26 August 2011; **B**, larva in an aquarium with vegetation associated from the collection site, photographed by F.F. Salles, 13 March 2015

*Nectopsyche fuscomaculata* Flint, 1983 

(Figs. 3, 4, 5, 6 and 7).

*Type locality*: Argentina, Pcia. Misiones, Arroyo Liso, 8 km W General Güemes.

*Holotype*: Male (pinned), collected on 19 Nov 1973, deposited in the National Museum of Natural History (NMNH), Washington, DC, USA. Catalog number: USNMENT01028361.

*Nectopsyche fuscomaculata* Flint, 1983: 73, figs. 254, 341 [male description]; Almeida and Marinoni 2000: 349 [distribution; biology]; Blahnik et al. 2004: 5 [distribution]; Paprocki et al. 2004: 12 [checklist]; Dumas et al. 2009: 368 [distribution]; Calor 2011: 322 [checklist]; Dumas and Nessimian 2012: 16 [distribution]; Souza et al. 2013: 6 [distribution]; Paprocki and França 2014: 58 [checklist]; Dias et al. 2015: 376 [distribution]; Holzenthal and Calor 2017: 324 [catalog]; Henriques-Oliveira et al. 2019: 285 [distribution]; Henriques-Oliveira et al. 2020: 44 [distribution]; Santos et al. 2022: 520 [distribution]; Bonfá-Neto et al. 2023: 316 [distribution]; Sganga et al. 2025: 9 [distribution]; Alvim et al. 2025 [distribution].

**Material examined.** BRAZIL: Espírito Santo, Sooretama, Rio São José, 19°07′33.1"S 40°14′26.1"W, elev. 25 m, 10.ix.2014, Pennsylvania light trap, 1♂ [alcohol], R.P. Constantino, col. (UFVB TR01083); same data, except 30.v.2011, 1♀ [alcohol] (UFVB TR01085); same data, except 23.iv.2015, 1 larva [alcohol] (UFVB TR01088); same data, except 13.i.2015, 1♂ [alcohol] (UFVB TR01082); same data, except 16.ii.2016, 1♀ [alcohol] (UFVB TR01084); same data, except 16.ii.2016, 1♂ [alcohol] (UFVB TR01086); same data, except 13.i.2015, 1♂ [alcohol] (UFVB TR01087).

**Description. Adult female** (in alcohol). Forewing length 7.1–7.4 mm (*n* = 2). Head pale yellowish (Fig. 3A); antennae pale yellowish (Figs. 3A, B); maxillary and labial palps pale yellowish (Fig. 3B); compound eyes width approximately 0.4 times the interocular distance (Fig. 3A). Thorax yellow; prothorax pale yellowish (Figs. 3A, B); mesothorax with pale yellowish to yellowish scutum, with 12 pairs of setal warts arranged in longitudinal rows; scutellum pale yellowish with two pairs of setal warts (Fig. 3A); metathorax yellowish (Fig. 3A); legs pale yellow (Fig. 3B), tibial spur formula 0–2-2. Forewing membrane hyaline without dark spots; area between veins Sc and R2 slightly thickened, resembling a pterostigma; apical forks I and V present; discoidal cell 0.8 times the length of the thyridial cell (Fig. 3C). Hindwing membrane hyaline without spots; radial and median veins atrophied; apical fork V present (Fig. 3C). Abdominal segment IX trapezoidal in lateral view (Fig. 4A); tergum IX with a pair of acrotergites (Fig. 4B). Tergum X triangular, apex tapered, with a small mesal notch (Fig. 4B). Appendage of segment X long and narrow, base slightly wider, setose (Fig. 4A, B). Digitate process thin, present in the mediolateral region, apically tapered, with one apical seta and a few basal and mesal setae (Fig. 4A). Valve rectangular, setose, associated with a long and narrow ventral sclerite (Fig. 4A, C). Vaginal apparatus (complex of spermathecal sclerites) consisting of partially to fully sclerotized sclerites connected by membranes, bearing a sclerotized ventral ridge with a longitudinal mesal cleft (Fig. 4A, C).

**Larva**. Body length 7.1–9.9 mm (mean = 8.4 mm; *n* = 7). Head and thorax setal pattern typical for the genus (Fig. 5E, F, J) (Holzenthal 1995). Head: light brown with numerous pale muscle scars (Fig. 5F, I); subocular ecdysial line present, in lateral view (Fig. 5I); labrum pale, subrectangular (Fig. 5F); mandibles light brown, short, wide, and excavated mesally, with short dark teeth (Fig. 5I); left mandible with 6 teeth and right mandible with 5 teeth; antenna pale, long and slender (Fig. 5F, I); ventral apotome short and subtriangular (Fig. 5J). Frontoclypeal apotome: anterior half with three pairs of circular muscle scars, first pair partially fused (Fig. 5E, F); medial region with a pair of diffuse light spots adjacent to the suture curve (Fig. 5E, F); posterior half with three longitudinally aligned muscle scars and a pair of small lateral marks (Fig. 5E, F). Parietal region: area above the subocular ecdysial line with approximately 12 muscle scars on each side, light marks varying in shape (Fig. 5F, I); gena and postgena, area below the subocular ecdysial line, with seven to nine muscle scars on each side (Fig. 5I, J). Thorax: pronotum light brown with brown spots, bearing anterior and lateral margins entire, not crenulate or dentate; anterolateral corners delimited by ecdysial line (Fig. 5E). Mesonotum with a pair of large medial sclerites, light brown with brown spots; mesonotal sclerite sa3 small, subtriangular and dark brown (Fig. 5E). Metanotum with a pair of tracheal gills present (Fig. 5A); metanotal sclerite sa3 small, oval, and weakly sclerotized (Fig. 5E); metasternum with a pair of long setae. Legs long and narrow, hind tibia not sub-divided (Fig. 5K, L, M); hind leg with two rows of long setae on femur and tibia; hind tarsus with rows of long dorsal setae and ventral rows of spine-like setae (Fig. 5M). Abdomen: Segment I with dorsal and lateral hump present (Fig. 5A); lateral hump sclerite with rounded anterior portion bearing fine microtrichia and a long, narrow, sclerotized, posterolateral extension, slightly curved ventrally (Fig. 5D). Lateral fringe not visible. Tracheal gills present, gill formula: I-0; II-3; III-2; IV-0; V-0; VI-0; VII-0; VIII-0; IX-0 (Fig. 5A). Abdominal segment X with lateral sclerite bearing robust, long setae (Fig. 5B); anal claw with two small accessory hooks (Fig. 5C).

**Larval case**. Length 13.4–20.5 mm (mean = 17.0 mm; *n* = 7). Straight cylinder, conical, and tapering posteriorly; composed primarily of small, thin plant fragments (likely fine adventitious roots of aquatic macrophytes) positioned obliquely and arranged in a roughly spiral pattern (Fig. 5G, H). Longer plant fragments are often attached longitudinally to the cases (Fig. 6A–C). Some examined cases consist of light-colored fragments, as seen in the associated larva (Fig. 5G, H), while others exhibit a predominance of small mineral particles in the anterior region (initial), likely built by early instars (Fig. 6B). Other cases show similar architecture but with a predominance of dark thin plant fragments and occasional small leaf fragments (Fig. 6E). In their early construction stages (anterior region), these cases are dominated by diatoms of the genus *Terpsinoë* Ehrenberg, 1843, interspersed with plant fragments and sparse mineral particles (Fig. 6D–G).

**Pupa**. Unknown.

**Remarks**. Due to the nature of the collection method for the sequenced material (specimens fixed in alcohol), the adults lack the original color pattern of the body and wing hairs and scale-like hairs. However, a live male of *N. fuscomaculata* with intact vestiture was recorded at the same collection site (Fig. 7A). The female of *N. fuscomaculata* described herein likely shares the same color pattern of the hairs as the male, as is commonly reported in the literature for other species of the genus where females exhibit the same color pattern as males.

According to the characteristics of the male genitalia, *N. fuscomaculata* can be placed in the *candida* or *bruchi* species groups based on the following characteristics (Flint 1968, 1983): preanal appendages simple and straight, and inferior appendages slender, straight or arched, lacking a large basal expansion and possessing a digitiform basoventral lobe. However, both the female and the larva exhibit similarities with species from both the *albida* (first mentioned by Ross 1944, p. 219, for males) and *candida* species groups.

Among the species with described females, *N. albida* (Walker, 1852) and *N. exquisita* (Walker, 1852) possess genitalia relatively similar to that of *N. fuscomaculata*. These two species, both with Nearctic distributions, are distinguished from *N. fuscomaculata* by the width of segment IX, which is narrower than long in the latter, whereas in the two mentioned species, segment IX is as wide as it is long. Additionally, the digitiform process in *N. fuscomaculata* is dorsally directed, while in the other two species, it is ventrally directed.

The larva of *N. fuscomaculata* is similar in its head spotting pattern (muscle scars) to *N. tavara* (Ross, 1944) and *N. gracilis* (Banks, 1901). However, they differ in the color patterns of the pronotum and mesonotum, the shape of the lateral hump sclerite, and the gill formula of the thorax and abdomen. In addition to these larval morphological differences, the case of *N. fuscomaculata* is distinguished from those of the other two species by being composed predominantly of small plant fragments, whereas the cases of *N. tavara* and *N. gracilis* are primarily constructed of sand grains (Daigle and Haddock 1981; Wiggins 1996).

**Fig. 8:**
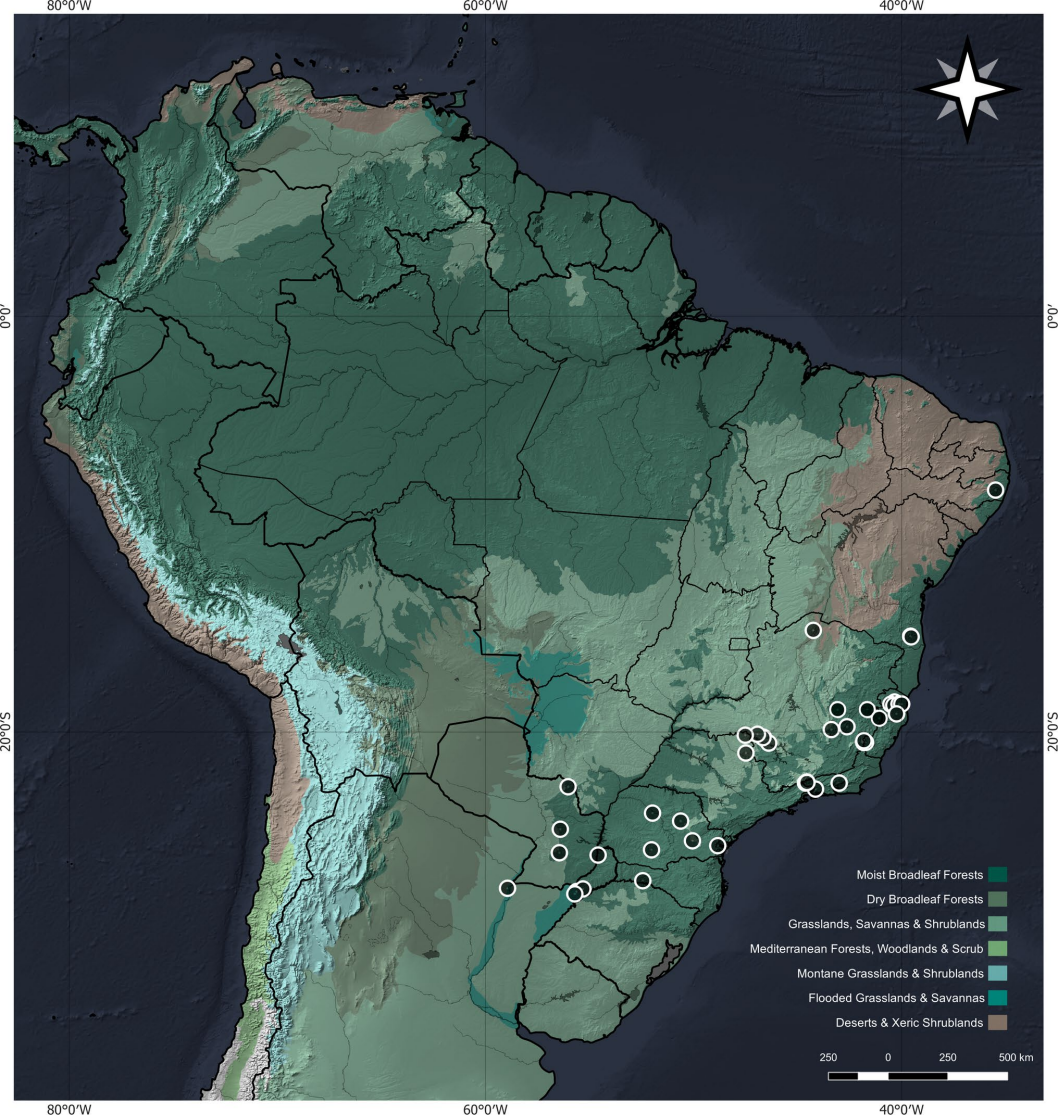
Map of the known geographical distribution of *Nectopsyche fuscomaculata*

**Distribution**. ARGENTINA, BRAZIL (Bahia, Espírito Santo, Minas Gerais, Pernambuco, Paraná, Rio de Janeiro, Santa Catarina, São Paulo), PARAGUAY. (Fig. 8).

## Discussion

*Nectopsyche fuscomaculata* has a wide geographical distribution, closely associated with the Atlantic Forest biome (Fig. 8). Only a few records of the species are located outside the Atlantic Forest domain or its transition zones. These records are located in the state of São Paulo, within the Cerrado domain, with many being in proximity to the Atlantic Forest, and one record from the floodplains of the Paraná River in Argentina.

In studies by Rafael and Py-Daniel (1989) and Flint (1991), the presence of females identified as potential *N. fuscomaculata* was recorded on Maracá Island, Roraima, in northern Brazil. However, specific identification could not be confirmed due to the denuded state of the body and wings, the loss of the characteristic vestiture pattern, and the lack of other known diagnostic characters for females at that time (Flint 1991). Therefore, in the current study, the record from Maracá Island is not considered part of the species' distribution due to these identification uncertainties and the significant geographical distance from its confirmed range.

The use of *Terpsinoë* diatoms in the early development of *Nectopsyche* cases had been previously documented for larvae of undetermined species in the southern United States of America (Wallace et al. 1976). Diatoms provide structural durability due to their siliceous frustules and regular shapes, which are essential for case construction, particularly during early instars. Additionally, the ridges and grooves present in *Terpsinoë* increase the surface area for silk attachment, enhancing the structural integrity of the cases (Wallace et al. 1976).

The application of DNA barcoding enabled the accurate association of the different life stages of *N. fuscomaculata*, including males, females, and larvae, while also revealing significant genetic variation in the COI gene within this lineage. Genetic distances of approximately 0.5% were observed between populations separated by ~ 730 km, specifically between Altinópolis, Rio Baguassu (RBCAD-2906), and Sooretama, Rio São José (TR01088). In contrast, the largest intraspecific divergence (4.3%) was detected between specimens from the same river in Sooretama (TR01087 and TR01088). Although this COI genetic distance within a single species may be considered high (Hebert et al. 2003), values exceeding 3% are relatively common in the order Trichoptera (e.g., Santos et al. 2016 for *Metrichia* Ross, 1938; Barcelos-Silva et al. 2018 for *Synoestropsis* Ulmer, 1905; Henriques-Oliveira et al. 2025 for *Triplectides* Kolenati, 1859).

This pattern indicates a lack of simple isolation-by-distance and suggests that genetic divergence is not strictly correlated with geographic separation in this taxon, as is commonly reported for freshwater insects (Schmidt et al. 1995; Bunn and Hughes 1997; Hughes et al. 1998). This may indicate cryptic lineage structure or restricted gene flow at a fine spatial scale (Wood and Resh 1991; Jackson and Resh 1998), possibly associated with microhabitat differentiation. Linearities of stream systems are hypothesized to provide a downstream succession of environmental conditions and communities, such as the selection of different substrates for net-building (e.g., Statzner et al. 2005; 2009; Statzner and Dolédec 2011), which could promote parapatric diversification (Statzner et al. 2010; Dijkstra et al. 2014).

Together, these molecular patterns provide a broader context for interpreting the observed morphological and ecological variation within *N. fuscomaculata*. Taxonomic observations revealed a wide range of materials used in the construction of *N. fuscomaculata* larval cases, highlighting the species' high plasticity in its building behavior. This plasticity is consistent with the molecular analyses, which showed high intraspecific genetic distances; this variation may be associated with the diverse larval habitats occupied by this species. As we obtained DNA sequences from only a single larva, it is not possible to verify whether the differences observed in the selection of case-building materials reflect the existence of distinct lineages. Regardless, no morphological differences were found among the larvae.

## Conclusion

This study contributes to reducing the Haeckelian gap in *Nectopsyche* by describing the larva and female of *Nectopsyche fuscomaculata*. It also represents the first work to address both interspecific and intraspecific molecular variation within the genus. Furthermore, it increases the number of species and DNA sequences available in genetic databases. Finally, the study highlights the importance of integrating molecular approaches into taxonomic research to improve our understanding of biological diversity.

## Supplementary Information

Below is the link to the electronic supplementary material.

Supplementary file1 (CSV 2 KB)

Supplementary file2 (CSV 8 KB)

Supplementary file3 (CSV 4 KB)

## Data Availability

All data generated or analysed during this study are included in this published article and its supplementary information files.
